# Monitoring antimicrobial usage in companion animals: exploring the use of the Danish VetStat database

**DOI:** 10.1186/s13028-022-00647-w

**Published:** 2022-10-17

**Authors:** Anne-Sofie Glavind, Amanda Brinch Kruse, Liza Rosenbaum Nielsen, Helle Stege

**Affiliations:** Department of Veterinary and Animal Sciences, Faculty of Health and Medical Sciences, University of Copenhagen, Groennegaardsvej 3, 1870 Frederiksberg C, Denmark

**Keywords:** Antimicrobial, Antimicrobial stewardship, Prescriptions, Register data, Surveillance systems, Validation, Veterinary sales data

## Abstract

**Background:**

In the Danish Veterinary Statistics Program, VetStat, sales data on medicinal products prescribed for veterinary consumption is collected. The Danish Food and Veterinary Administration (DVFA) manages the database and each purchase contains detailed product-specific information linked with a species-specific ID. National surveillance systems are also implemented or being developed in the other European Union Member States. By 2029, all Member States are required to report data on antimicrobial usage for companion animals to the European Medicines Agency. This study aimed to assess the challenges encountered when using the VetStat database to quantify antimicrobial use in Danish companion animals. Raw VetStat data were propagated by the DVFA and originated from veterinary practitioners and Danish pharmacies.

**Results:**

Comprehensive estimates of antimicrobial use in Danish companion animals were not readily available due to database construct. Antimicrobials sold for use in companion animals (linked to a companion animal ID) comprised a large number of products licensed solely for horses or livestock, while data assigned a replacement code encompassed both topical and peroral antimicrobials licensed for companion animals. Additionally, antimicrobials sold from pharmacies to veterinary practitioners presented the biggest challenge in data retrieval and validation. Treatment data are only transferred to VetStat through the billing systems when Danish veterinarians are treating livestock, but not companion animals. Information on products sold for in-house use in companion animals is only available from pharmacy records without a species-specific ID. As a result, parenteral antimicrobials with multi-species authorization utilized by small animal veterinary practitioners are not accounted for in the overall estimate for companion animals.

**Conclusions:**

Owing to the database structure and requirements for data entry, antimicrobial use in companion animals is an approximation. The actual consumption may be significantly higher than what is currently calculated from the database, as the majority of parenteral products are not included. Consumption data can be measured more accurately provided treatment data from veterinary practitioners in small or mixed practices are transferred to the database through the billing system. This would equal the legal requirements for Danish veterinary practitioners treating livestock.

## Background

Antimicrobial resistance (AMR) is a global public health threat that requires monitoring of usage patterns in both human and veterinary medicine [1–3]. The selective spread of AMR-resistant pathogens from livestock to humans is a primary concern [2–4], but companion animals are increasingly recognized as potential reservoirs [5–10]. Advanced specialization in small animal medicine has generated a demand for larger referral hospitals allowing for longer-term admissions, and treatment of geriatric or critically ill patients, giving rise to more high-risk veterinary patients in the veterinary field [11]. Nosocomial infections and clinical outbreaks of AMR-resistant pathogens can reduce the treatment efficacy [9, 10] and compromise animal welfare. The zoonotic properties in many of the pathogens are a potential workplace hazard for veterinarians, whilst the close cohabitation between owners and their pets facilitates the spread of AMR genes and pathogens outside hospital settings [5, 10–12].

The use of critically important antimicrobials (CIA) for human medicine in the veterinary field is another concern, drawing attention to the stewardship of these in small animal medicine [5]. Current classification by the World Health Organization (WHO) has ranked 3rd to 5th generation cephalosporins, polymyxins, quinolones, glycopeptides, and macrolides as those of highest priority [13]. In Europe, many of these are commonly applied to treat companion animals [10], and in Denmark, almost all prescriptions of fluoroquinolone for veterinary purposes are made by small animal practices [14].

In the European Union (EU), The European Surveillance of Veterinary Antimicrobial Consumption (ESVAC) provides an overview of AMU in the veterinary field. Data on sales of veterinary antimicrobials from each member state (MS) are published in yearly reports as milligrams per Population Correction Unit (mg/PCU) [15, 16]. However, consumption data for companion animals is not yet included since data is not available in all MS [15].

Reporting of total consumption in the veterinary field is often limited to national surveillance schemes, and at the time of writing, 16 countries had systems in place to monitor AMU in livestock [17]. The systems apply different indicators and metrics [18], and only a fraction includes some variations of usage data on companion animals with data originating from pharmacy recordings, voluntary admissions, selected clinics, or surveys [19].

In Denmark, a national database (the Veterinary Statistics program, VetStat) collects sales data on drugs prescribed for veterinary consumption from three main sources; pharmacies, feed mills, and veterinarians [20–22]. Veterinary antimicrobials are available by prescription-only [21], and a purchase is linked with information on prescribing veterinarian, the reporting pharmacy, Anatomical Therapeutic Chemical (ATC) codes or ATCvet-codes, and a species-specific ID. Sales recorded without a species-specific ID are listed with a replacement code (animal species code 0). Antimicrobials are sold from Danish pharmacies to veterinary practitioners or directly to farmers or pet owners on prescription. Products purchased by veterinary practitioners are often recorded in the VetStat database with only a veterinary practice ID. Danish veterinarians treating livestock are obliged to record each treatment in VetStat with information on species, age, and disease group. The veterinarian must also register a Central Husbandry Register code (CHR-number), which refers to the location and species of the farm property [20, 21]. Data is often transferred automatically to VetStat from the electronic billing system of the veterinary practice. Veterinarians treating companion animals are not subject to the same requirements, and there is no legal obligation for veterinarians to transfer data from Danish companion animals to VetStat [21, 22], which means that only sales from pharmacies to pet owners are linked with an animal species code.

The governmentally supported Danish Integrated Antimicrobial Resistance Monitoring and Research Program (DANMAP) publish yearly reports on AMU in Danish animals based on VetStat data [23]. Given the challenges connected to animal species codes omitted in part of the VetStat data, DANMAP has adopted a method of retrieving sales data on companion animals from several different VetStat tables [23–25]. Antimicrobials without an animal species code are allocated to companion animals based on product license (dogs and cats only) or preparations (tablets, capsules, ear- and eye drops). Additionally, oral preparations recorded as prescribed under the companion animal species code are omitted provided the products are licensed solely for livestock or horses. Parenteral preparations with multi-species authorization recorded without an animal species code are not assigned to companion animals [24]. This suggests that several obstacles emerge in retrieving accurate sales data for companion animals.

By 2029, all MS are required to report national AMU in companion animals [6, 26]. In addition, several countries are currently in the process of establishing national surveillance systems, with varying inclusion of animal species [17]. To comply with the forthcoming EU requirements, it is necessary to strengthen and validate existing national systems and to test the usability of each database in retrieving valid information on usage data on companion animals.

Therefore, the present study aimed to assess the usability of the VetStat database for estimating national sales of antimicrobials in Danish companion animals and to present data in mg/PCU as suggested by ESVAC [15]. More specifically the objectives were to: (1) quantify the total sales of AMs in Danish companion animals (dogs and cats) in 2018 from data available in the VetStat database, (2) stratify sales data based on antimicrobial classes, preparations, and licenses, (3) use the national sales data to calculate mg/PCU, and (4) describe and discuss the main challenges in using VetStat data to quantify total AMU in companion animals.

## Methods

### Descriptive analysis

Data were extracted from VetStat in august 2019 and descriptive analysis was performed in R (version 4.0.3 of 2020–—The R Foundation for Statistical Computing) and in Microsoft Excel.

### Extracting and processing VetStat data

To estimate the total amount of AMs sold for use in Danish companion animals in 2018, raw data from VetStat were extracted in august 2019 by the DVFA. Information on products purchased at the pharmacy (by animal owners or veterinary clinics) and products used by veterinary practitioners treating livestock are automatically transferred to the VetStat database. Data used in the present study thus originated from Danish pharmacies and veterinarians.

The methodology presented in DANMAP [24] was applied in the present study as a basic principle for the examination of VetStat data. In DANMAP [23], consumption data related to companion animals were compiled from AMs with no specified animal species (animal group code 0) provided that license, preparation, or concentration were applicable to companion animals and from products prescribed for companion animals with the exclusion of oral AMs licensed solely for horses and livestock. DANMAP does not allocate parenteral antimicrobials with multi-species approvals recorded without a specified animal species [24].

**Fig. 1:**
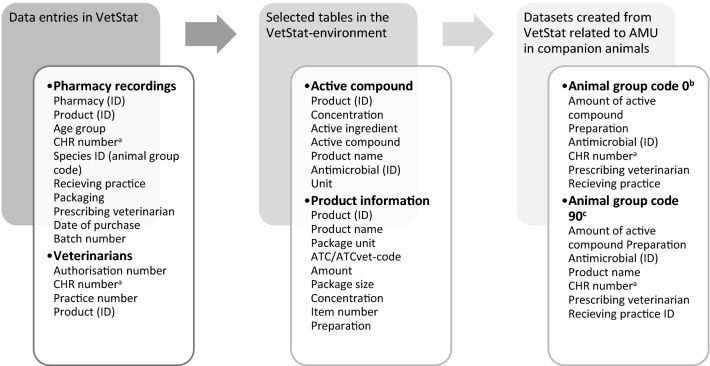
Data entries and product details from selected tables in the VetStat environment. A simplified version of raw data in the VetStat environment. Data entries from pharmacies and veterinary practitioners are combined with variables from various tables in the VetStat environment to create datasets with information on antimicrobial use in companion animals. The datasets are created by extracting sales data recorded with either a companion animal ID or a replacement code. ^a^Central Husbandry Register. The CHR number refers to the location, size, and type of a specific farm property ^b^Corresponds to a species-specific ID ^c^Anatomical Therapeutic Chemical classification system/ Anatomical Therapeutic Chemical classification system for veterinary medicinal products ^d^Corresponds to records without a species-specific ID (replacement code). ^e^Corresponds to records with a species-specific ID (animal group code) assigned to companion animals

In the present study, two datasets were created from the tables in the raw VetStat data; one covering AMs sold directly from pharmacies for use in companion animals (recorded on animal group code 90) and another covering AMs sold from pharmacies with no specified animal species (recorded on animal group code 0, i.e. a replacement code). Data entries are accepted in the database regardless of animal species. Consequently, AMs licensed for one animal species may erroneously be recorded under a conflicting animal group code. Each dataset thus contained the same variables (product ID, product name, prescribing veterinarian, receiving practice, CHR number, and amount of active compound) to enable examination of possible errors (Fig. 1). Consumption data were calculated as a weight-based unit (kg active compound). Information on product license was included by official product descriptions and approvals [27] validating February 2020. Both datasets were subdivided by license and preparation.

Because the database allows products to be recorded under conflicting animal group codes, AMs from animal group code 90 were excluded provided license or preparation indicated that the products in question were recorded under an erroneous animal group code. In the present study, intramammary, intrauterine, and topical preparations for livestock were excluded in addition to oral preparations licensed for horses or livestock. This differs slightly from DANMAP [23].

A similar procedure was applied to the data covering animal group code 0. Antimicrobials were transferred to the remaining data from animal group code 90, provided that license or preparation were pertinent to companion animals. The quantity of AMs sold for use in Danish companion animals was thus a calculated estimate, referred to hereafter as AMU_calc_.

Furthermore, each dataset was examined for the presence of invalid practice numbers and omitted veterinary authorization numbers to evaluate product traceability.

AMU_calc_ covering 2018 was then stratified according to antimicrobial classes (aminoglycosides, amphenicols, cephalosporins, fluoroquinolones, lincosamides, macrolides, penicillins, pleuromutilins, sulphonamides, trimethoprim, and tetracyclines), preparation (parenteral, oral powder, tablets, oral paste, ointments, eye, and ear drops), and specific product name.

### Parenteral antimicrobials

Parenteral antimicrobials sold from pharmacies for use in veterinary clinics were examined to assess if data alone were sufficient to indicate the quantity used for companion animals. New datasets were created from the raw VetStat data. The datasets covered parenteral AMs sold from pharmacies to veterinary practitioners (recorded with a veterinary practice number, but no CHR number) and parenteral AMs used by veterinary practitioners to treat livestock (recorded with a CHR number and livestock species ID and transferred to VetStat through the billing system). Sales and consumption data were calculated as a weight-based unit (kg active compound), and products with multi-species authorization were identified. The difference between the amount of parenteral AMs sold from pharmacies to veterinary practitioners and the parenteral AMs recorded by veterinary practitioners treating livestock was calculated. The difference between sold and consumed amount thus covered parenteral AMs sold to small animal veterinary practitioners, wastage, or products still on the shelf. It may indicate the magnitude of parenteral AMs administered for in-house treatment of companion animals.

### Calculating the population correction unit

National sales data from each of the ESVAC participating countries are harmonized by including the population at risk in the denominator. The metric applied is the “population correction unit (PCU)”, which serves as a standardized measure for the population potentially treated in each MS [15]. In the present study, PCU for Danish companion animals was calculated using a simplified version of the PCU for livestock [15]. Only dogs and cats are considered, as the significance of rodents and birds is negligible in small animal practices in Denmark.

Calculation of the Population Correction Unit (PCU) for Danish companion animals:

$$PCU_{Companion \,animals} = \left( {n_{d} *AW_{d} } \right) + \left( {n_{c} *AW_{c} } \right)$$

where n is the estimated population size and AW is the average standard weight (in kg) at the time of treatment for dogs (d) and cats (c) respectively.

In the present study, PCU_Companion animals_ were calculated using standard live weights of dogs (20 kg) and cats (4 kg) [25] and the individual population size of Danish dogs (810,000 heads) and cats (730,000 heads) [28].

The overall national consumption can thus be further approximated by adding sales data from companion animals (peroral AMs) and biomass (PCU_companion animals_) separately to that of livestock already presented in the ESVAC reports.

Calculation of mg sold per PCU for Danish livestock and companion animals:

$$mg/PCU_{national} = \frac{{national \,sales\, of\, antimicrobials \left( {mg} \right)}}{{PCU_{livestock} \left( {kg} \right) + PCU_{companion \,animals} \left( {kg} \right)}}$$

## Results

### National antimicrobial sales data

Sales data assigned a companion animal ID (animal group code 90) covers sales of AMs from pharmacies for intended use in companion animals (mainly products purchased by the pet owner). The consumption amounted to 515 kg active compound in 2018. However, 53% (275 kg active compound) were products licensed solely for use in livestock or horses. Because the database accepts sales data even though the license or preparation does not match the assigned animal group code, the products in question were regarded as registration errors, and thus not prescribed for companion animals. The products included oral paste licensed for horses (241 kg active compound), intramammary and intrauterine AMs licensed for cattle only (< 1 kg active compound), AMs solely for aquaculture (1 kg active compound), parenteral preparations licensed for horses or livestock (3 kg active compound), topical sprays for livestock (< 1 kg active compound), and feed additives for livestock (30 kg active compound). After deducting sales data inconsistent with AMU in companion animals, the remaining amount of AMs recorded on animal group code 90 was more than halved (240 kg active compound remaining).

Sales data assigned a replacement code (animal group code 0), hence recorded in the database without a species-specific ID, covers sales of AMs from pharmacies to veterinary practitioners and occurrences where a species-specific ID has been erroneously omitted. This amount was 5956 kg active compound in 2018.

Parenteral preparations represented more than half (3451 kg active compound), and the majority were products with multi-species authorization (2609 kg active compound). The remaining products recorded without a species-specific ID comprised peroral and topical antimicrobials. From license and preparations (tablets, ointment, ear- and eye drops, or licenses solely for dogs and cats), a total of 706 kg active compounds were identified and assigned to companion animals.

Hence, aggregated sales data from the different VetStat tables provided the source for a calculated estimate of AMU in Danish companion animals, and AMU_calc_ amounted to 946 kg active compound in 2018. AMU_calc_ is clearly an approximation and does not include parenteral AMs with multi-species approval assigned a replacement code.

Veterinary practice and authorization IDs provided information on presumed product distribution. The majority of AMs assigned a specific companion animal ID were sold from Danish pharmacies to pet owners from veterinary prescriptions (no assigned CHR number in the data). Less than three kg active compound was sold directly to veterinary practitioners or farmers (only a veterinary practice ID or CHR number is available in the data).

More than 90% of AMs assigned a replacement code (no species-specific animal ID) were products sold from pharmacies directly to veterinary practitioners (practice ID recorded). A small fraction was sold to farmers from veterinary prescriptions (CHR number recorded) or to individual veterinary practitioners (veterinary ID).

### Parenteral antimicrobials

Examination of parental antimicrobials assigned a replacement code revealed that products with multi-species approval purchased for use in veterinary clinics greatly exceeded the amount retrieved in the veterinary recordings, i.e. treatment of livestock recorded by the veterinary practitioners.

Deducing consumption data recorded by veterinary practitioners from the pharmacy sales of antimicrobials directly to veterinary clinics yielded a difference of 552 kg active compound. This amount covers parenteral AMs that have not been used to treat livestock and therefore contains the number of products with multi-species approvals used for the in-house treatment of companion animals. It covered purchases from 697 veterinary clinics (697 different veterinary practice IDs), but to which extent the amount also covered wastage or wrongly omitted recordings of CHR numbers could not be explained solely from the VetStat data.

A comparison of 2018 with previous years revealed that the quantity varied from 128 kg active compound in 2016 to 583 kg active compound in 2017.

### Preparations and antimicrobial classes

By reference to AMU_calc_, antimicrobials on peroral preparations (mainly tablets and capsules) were by far the most applied route of administration (915 kg active compound) for companion animals in 2018, followed by parenteral (20 kg active compound) and topical preparations (11 kg active compound).

In Table 1, AMU_calc_ is further subdivided into antimicrobial classes. Extended-spectrum penicillins represented more than two-thirds in 2018 (677 out of 946 kg active compound). Cephalosporins accounted for 97 kg active compound, lincosamide, and tetracycline for 64 and 24 kg active compound, respectively. The remaining 84 kg active compound was distributed on amphenichols, aminoglycosides, fluoroquinolones, macrolides, simple penicillins, sulfonamide, trimethoprim combinations, tiamulin, and *others* (fusidic acid, sulfasalazine, and metronidazole). From AMU_calc_, 12 kg active compound (1.3%) were antimicrobials categorized by WHO [13] as the highest prioritized CIAs. Administration of cephalosporins for companion animals was mainly 1st generation cephalosporin for peroral administration (96 kg active compound), whereas 3rd and 4th generation cephalosporin accounted for 1 kg active compound. Fluoroquinolones and macrolides were 11 and < 1 kg active compound, respectively.

**Table 1:** Calculated estimate (AMU_calc_) of national sales of antimicrobials for use in Danish companion animals (dogs and cats) in 2018

Antimicrobial class	Preparations^1^			Total
	Peroral	Topical	Parenteral	
Penicillin (ext.)^a^	675	–	2	677
Penicillin (sim.)^b^	4	–	16	20
Cephalosporin (1st gen)	96	–	–	96
Cephalosporin (3rd & 4th gen)	–	–	1	1
Lincosamides	64	–	–	64
Tetracycline	23	< 1	–	24
Amphenicols	–	< 1	–	< 1
Aminoglycosides	–	1	< 1	1
Quinolones^c^	11	< 1	< 1	11
Macrolides	< 1	–	–	< 1
Sulfonamide^d^	1	–	< 1	2
Other^e^	40	8	–	48
Polymyxin	–	< 1	–	< 1
Total	915	11	20	946

^a^Penicilins with extended-spectrum (amoxicillin-clavulanate acid)

^b^Simple penicillins

^c^Specified values comprise fluoroquinolones

^d^Including sulfonamide/trimethoprim combinations

^e^Fucidic acid, sulfasalazine, and metronidazole

^1^Active compound (kg)

### Calculating the population correction unit

The population correction unit for Danish companion animals (PCU_companion animals_) calculated in the present study was 19,120 tonnes. As a result, the approximated contribution from Danish companion animals to the overall national consumption amounts to 915 kg active compound (peroral AMs) and 19,120 tons of live weight.

In 2018, the magnitude of veterinary AMs for Danish livestock amounted to 38.2 mg sold per PCU [15]. The metric covers sales of 93.6 tonnes of active compound and a PCU of 2,446,700 tonnes. Adding the approximated contribution from companion animals brings the Danish consumption (mg/PCU_national_) to 38.3 mg sold per PCU.

### Data issues

Applying VetStat data as a means to estimate total sales of AMs in companion animals presented a number of challenges. The challenges, which are summarized in Table 2, refer to the extraction, analysis, and validation of data.

**Table 2:** Challenges encountered in using VetStat data to estimate antimicrobial sales data in Danish companion animals

Data entry	Data extraction	Analysis and interpretation of raw data
Only treatment data from livestock are transferred to VetStat	Data from several animal group codes may be necessary when assessing AMU^a^ in one species	Knowledge of the nature of data and construction of the database is necessary
Missing or faulty recordings of Danish veterinary practice or authorization numbers	Raw data from a specific animal group code may contain sales data for other species than those relevant for the group code in question	Calculation of sales data from several animal group codes may be necessary
The database does not receive treatment data for companion animals		Interpretation of raw data from only one group code may lead to faulty conclusions
Information on animal species (animal group code) are not recorded for antimicrobials sold to veterinary practitioners		

^**a**^Antimicrobial use

The main challenge in extracting total sales of veterinary AMs for companion animals was encountered in the data covering sales of antimicrobials to veterinary practitioners. Products sold from pharmacies to veterinary practitioners and used for the treatment of livestock were readily available by the veterinary recordings, but veterinary practitioners treating companion animals are not obliged to transfer data to VetStat, so the database does not contain any treatment data for Danish companion animals.

Consequently, products sold from pharmacies to veterinary practitioners are often recorded without a species ID (hence assigned the replacement code 0). Each sale is recorded with a veterinary practice ID, but the database does not cross-reference with the practice type (small, large, or mixed practice). Consumption data for companion animals, therefore, relies on a calculated estimate (in the present study referred to as AMU_calc)_ with sales data retrieved and calculated from both animal group codes 0 and 90. If sales data were extracted solely from animal group code 90, companion animals would mainly account for pharmacy recordings of products sold to pet owners (i.e. veterinary prescriptions). Peroral and topical preparations distributed by veterinary practitioners (706 kg active compound in 2018) would be unaccounted for. It complicates data extraction and increases the risk of erroneous data analysis. This is in particular demonstrated by the lack of species-specific ID in parenteral AMs used in-house by small animal veterinary practitioners (recorded with only a practice number), which complicates any register data-based estimate of multi-species approved AMs for companion animals.

General data validation also proved challenging. From animal group code 0, 10.5% of the veterinary practice numbers were invalid (less than four digits) and a small segment of entries (approximately 1 kg active compound) was recorded with only product-specific information, but no CHR number, veterinary, or veterinary practice ID.

## Discussion

VetStat data related to companion animals is currently used as the data source for the yearly DANMAP reports. However, due to the EU regulation, it is expected that the focus will be directed more towards AMU in companion animals. This may lead to an increased interest in VetStat data on companion animals from both the veterinary authorities, researchers, and other stakeholders.

In this study, the method of estimating AMU in Danish companion animals presented in DANMAP [24] was tested. Sales data from 2018 were reviewed with a thorough evaluation of specific products recorded both with and without an animal group code related to companion animals, and the study depicts the large disparity of recorded AMs for companion animals between animal group codes 0 and 90. AMU_calc_ is presented in kg active compound. Due to sales from pharmacies to veterinary practitioners often being recorded without an animal species code, parenteral antimicrobials from the pharmacy and veterinary recordings were examined to present an estimate of antimicrobials that could have been used in-house for companion animals. Furthermore, the study identified several challenges encountered when using register data such as the Danish VetStat database.

### Quantification of national antimicrobial sales data

An objective of this study was to apply national sales data from the VetStat database as a means to estimate total sales of AMs in Danish companion animals. The VetStat database provided detailed information on overall AMs in both animal categories (i.e. group codes 90 and 0) selected for the present study.

The various tables in the VetStat environment enable national sales data to be extracted as prescription data recorded under a specific animal group code [22], which provides an easy overview of records for selected animal species. One approach to estimate AMU in companion animals would therefore be to extract sales data recorded solely on animal group codes related to companion animals, but the present study made it evident that it would generate insufficient estimates. These findings support the method applied in DANMAP and emphasize the necessity of retrieving usage data from several tables and animal group codes, although this may seem redundant in a database that separates data by animal species.

An important finding in this study was how oral preparations licensed solely for companion animals were recorded in the database. A skewed distribution between animal group codes 0 and 90 was evident as the amount of peroral preparations, mainly tablets, recorded on animal group code 0 exceeded the total amount recorded at animal group code 90 with more than 300 percent. The products were recorded with a veterinary practice ID, hence sold to veterinary practitioners, which emphasizes the issue of traceability occurring because small animal practitioners are not obliged to transfer data on AMU to VetStat. Similar challenges may be encountered in other national surveillance systems built with the primary aim of recording consumption data for livestock.

The difference in total recorded amount between animal group codes greatly increases the risk of underestimating the actual AMU for companion animals provided data from animal group code 90 are used solely to quantify total AMU. Thus, the validity of results based solely on records from the VetStat database will rely greatly on the researcher’s knowledge of the nature of the data and the structure of the database itself.

However, a database such as the Danish VetStat database with records of all products sold for use in companion animals is a vital resource in AM surveillance since it also includes preparations licensed for humans only. Other national monitoring systems do not include preparations licensed solely for humans in the surveillance of sales data for companion animals or total AMU is measured based on peroral preparations only [29, 30]. Overall, this entails an imminent risk of underestimating the overall veterinary AMU. In the VetStat database, 58 kg active compound of parenteral AMs licensed for humans were recorded without an animal species, however, it is still included in the overall national estimate from DANMAP [23].

### Parenteral preparations

The study aimed to quantify the total sales of AMs for Danish companion animals. Therefore, parenteral AMs recorded without an animal species ID (replacement code 0) were examined to assess, whether VetStat data alone could elucidate the scope of products used in-house for companion animals by veterinary practitioners. Deduction of veterinary consumption data from pharmacy sales records provided an estimate of 552 kg active compounds of parenteral products with a multi-species license sold to 697 different veterinary practices. A valid estimate of the actual amount used to treat companion animals could not be inferred from data alone, since the amount most likely also covered unused products, wastage, or omitted usage recordings from large animal veterinarians. However, these findings strongly emphasize that allocation of the parenteral AMs in question to the correct animal species is crucial in order to produce valid estimates of companion animal sales data in the future.

The Netherlands Veterinary Medicines Authority (SDa) has addressed similar issues in estimating veterinary AMU based on sales and consumption data. Due to multi-species authorization, approximately 17 kg active compound could not be assigned a correct animal species. The SDa conducted a questionnaire survey among 100 veterinarians to elucidate the correct animal species to which the AMs were used [31]. Plausible solutions under Danish settings could be data validation through questionnaires distributed to each of the veterinary practitioners with purchases of the products in question. However, a solution like that is labor-intensive and dependent on a high response rate. A more sustainable solution would instead be found by including small animal veterinarians in the statutory data reporting to VetStat.

### Antimicrobial classes and preparations

Products from the beta-lactam class were the most commonly prescribed AM for companion animals. The largest quantity was extended-spectrum penicillins, making up 72% of the total amount. Simple penicillins accounted for 2% and cephalosporins for 10%. This preponderance compares with data from Europe and the UK [29, 30, 32–35]. Lincosamides accounted for 8% of the total prescribed amount, and tetracyclines and fluoroquinolones for 3 and 1 percent respectively. In regards to lincosamides and fluoroquinolones, the results from the present study are in agreement with reports from Norway and Finland [29, 30], but Denmark differs in the use of tetracyclines when compared with neighboring countries. The larger amount of tetracyclines recorded in Denmark may be linked to those preparations often being licensed for human consumption, which contrary to VetStat, is not included in the estimates from Norway and Finland [29, 30]. Results from Spain and Italy show a different use of fluoroquinolones, as those preparations are reported as the most commonly used following penicillins and cephalosporins [34, 35].

AMs sold for use in Danish companion animals were most frequently for oral administration. The same is evident in other European countries [29, 30, 33, 34, 36].

### Applied metrics

In VetStat, sales data for companion animals are presented as a weight-based indicator (kg active compound). There is currently no international consensus on appropriate metrics to report AMU or accommodate comparison of exposure data. Numerous metrics are proposed in the literature, but a lack of standardization may compromise the comparability of data [18, 37, 38]. Weight-based indicators are currently applied by DANMAP [23] and EMA [15] to describe trends in sales data stratified by nation or species. Presenting the weight of active compounds (mg or kg) alone or adjusted by a standardized correction unit (PCU) makes for an intuitive measure of overall sales data [39]. Although it does not provide information on drug potency [39] it does elucidate possible interspecies differences in AMU. Several dose-based metrics are applied in research or surveillance of AMU in livestock, with the “average defined daily dose” (DDDvet) presented in the ESVAC reports and “animal daily doses” (ADD) and the percentage of animals treated per day (ADDs per 100 animals per day) in Denmark [15, 23]. Such metrics require accurate estimates of population size, which is currently not available for companion animals in many MS.

Several studies have described AMU in small animal practice by quantifying usage data in selected clinics [34, 36, 40–43]. This enables detailed information on the population at risk, including weight, but does not yield information on the overall population size. The current method in [15] for quantifying AMU in livestock production is the use of a Population Corrected Unit (PCU) as the denominator. Companion animals are not yet included due to difficulties with consistent and valid information on population size and average weight from each MS [44]. The denominator presented in this study is a rough estimate, which emphasizes the challenges in achieving a valid estimate of the population at risk. The challenges of obtaining valid estimates from the currently available data are further reinforced by the highly approximated contribution of Danish companion animals to the overall national sales of AMs. For now, it is currently not possible to define the consumption of parenteral AMs in Danish companion animals, which means that valid comparison across member states remains limited. Transfer of usage data from in-house treatments of companion animals to VetStat and other national databases through the billing system could ensure that Denmark and other MS can provide valid data on AMU for companion animals to the ESVAC as is expected from 2029 [6].

### Data issues

The primary purpose of the present study was to test the usability of the VetStat database to quantify AMs sold for use in Danish companion animals. The VetStat database proved useful, as data is easily accessible, detailed, and does not require extensive time to collect. However, data processing presented several challenges. The challenges arose in extracting a complete dataset covering the actual sales data for companion animals. In the present study, data from animal group code 90 proved incomplete, as it also contained preparations licensed for livestock only. To achieve an approximated estimate of total sales data for companion animals, a large amount of sales data had to be retrieved from another group code in the database. Although in this case, AMU_calc_ is incomplete, as it does not contain parenteral AMs used for in-house treatments by veterinary practitioners. In the present study, antimicrobials were grouped according to product preparation and license. Each product license was recorded and coded manually, as it is not part of the VetStat environment. To reproduce the study on a yearly basis, or if similar methods are used for retrospective studies, updating product licenses manually will be very labor-intensive.

The present study illustrates that the main challenge is how data on antimicrobials for companion animals are recorded and stored in VetStat. AMs prescribed by the veterinarian for intended use in companion animals are most often purchased at the pharmacy by the owner of the animal. Here, the animal species and veterinarian’s authorization number are recorded in VetStat [20, 21] under animal group code 90. However, AMs sold to veterinary practitioners for use in-house are rarely recorded with a species-specific ID since no distinction is made by practice type. Most often, these products are only recorded with a veterinary practice number and the consumption data are subsequently transferred through the billing system when veterinarians treat livestock. This implies that parenteral antimicrobials used to treat companion animals, either in small animal or mixed practices, and peroral antimicrobials (often tablets) sold to the owner directly from the veterinary practice will only appear in VetStat at the pharmacy records (under the replacement code 0). For now, only peroral antimicrobials from the replacement code are transferred manually to companion animals.

A permanent solution to this issue would be to make it mandatory for Danish veterinarians in small animal and mixed practices to record each in-house treatment of companion animals in VetStat with species-specific information similar to what applies to veterinarians treating livestock. A change of this magnitude would require that the individual billing programs used in small animal practices are set up to automatically transfer data to the VetStat database. This would ensure that parenteral AMs used for in-house treatments of companion animals would be accounted for as well as peroral antimicrobials sold by veterinary practitioners.

A change of this magnitude will also allow researchers to extract valid estimates on AMU for companion animals from the veterinary recordings in the VetStat database. Transferring consumption data from small animal veterinary practitioners to the VetStat database will provide essential knowledge on the actual use of AMs in companion animals. It may also alleviate the challenges related to continuous monitoring of the population size and weight of the domesticated population of dogs and cats.

The present study also identified multiple data entries of peroral AMs with invalid veterinary practice IDs making it impossible to ensure valid product traceability. These findings demonstrate a profound issue related to the entry of sales data into the VetStat database as well as the indication of erroneous entries made at the pharmacy at the time of purchase. Entries of invalid veterinary identification ID or animal species ID have also been addressed and discussed by [45] in connection with AMU in Danish livestock. AMs prescribed by veterinary practitioners for use in livestock production are also purchased at the pharmacy. Pharmacies, therefore, partake an important role in entering valid and correct information on the sales of all veterinary antimicrobials to the VetStat database, but because the pharmacist conducts recordings manually [45], errors or omissions in the information entered in the VetStat database can occur.

Outlines of national medicinal databases and lessons learned from their use may serve as a prerequisite for how other databases can be designed and implemented. The importance is emphasized by the requirement for EU Member States to report valid estimates of AMU in companion animals. A database like the Danish VetStat is advantageous because all sales of antimicrobials for veterinary consumption are recorded and accounted for. Although the discrepancy between how detailed usage data in livestock is recorded and the fact that usage data in companion animals is not accounted for demonstrates that improvements are still required. If Denmark extends the mandatory transmission of consumption data to small animal veterinarians, it will also facilitate that outcomes of national measures and guidelines on prudent use of AMs in small animal medicine can be monitored from secondary data.

## Conclusions

Owing to the structure of the VetStat database, quantification of AMU in Danish companion animals is an approximation. The actual consumption may be significantly higher than what is currently calculated from the database, as the majority of parenteral products used in-house by small animal veterinarians are not included. National AMU in companion animals can be measured more accurately provided treatment data from veterinary practitioners in small or mixed practices are transferred to the database through the billing system. This will equal the legal requirements for Danish veterinary practitioners treating livestock.

Transfer of usage data from in-house treatments of companion animals to VetStat will also ensure that Denmark can provide valid data on AMU for companion animals to the ESVAC as is expected from 2029 since population size and standard weight remain uncertain.

## Data Availability

The datasets used and analyzed during the current study are available in anonymized form from the corresponding author on reasonable request. The authors declare that the research was conducted in the absence of any commercial or financial relationships that could be construed as a potential conflict of interest.

## Abbreviations

AM: Antimicrobial
AMU: Antimicrobial use
AMU_calc_: A calculated estimate of antimicrobials sold for use in Danish companion animals
AMR: Antimicrobial resistance
ATC: Anatomical therapeutic chemical classification system
ATCvet: Anatomical therapeutic chemical classification system for veterinary medicinal products
CIA: Critically important antimicrobials for human medicine
CHR: Central husbandry register code
DANMAP: Danish integrated antimicrobial resistance monitoring and research program
DVFA: The Danish food and veterinary administration
ESVAC: The European surveillance of veterinary antimicrobial consumption
MS: European member states
PCU: Population correction unit
